# Care staff perspectives of clinically meaningful outcomes of supportive care programs for persons with dementia in Kentucky: A qualitative multiple case study

**DOI:** 10.1002/alz.090446

**Published:** 2025-01-09

**Authors:** Alaine E. Reschke‐Hernandez, Martina Vasil, Emma G. King, Carson Woolums

**Affiliations:** ^1^ University of Kentucky, Lexington, KY USA

## Abstract

**Background:**

Emerging research suggests that complementary and supportive care programs, such as music therapy, show positive short‐term impacts (e.g., purposeful engagement, positive emotions) on persons with dementia who live in care facilities. However, barriers exist in understanding clinically meaningful outcomes (i.e., noticeable, valuable changes in a person’s life) in more advanced dementia and reliably documenting enduring outcomes within demanding care structures. This research aims to address these gaps by exploring clinically meaningful outcomes of such nonpharmacological programs from the perspective of clinical care staff.

**Method:**

Because healthcare policies vary across the United States, this qualitative multiple case study is bound to Kentucky. This focus facilitates in‐depth analysis and description in the context of the state healthcare system. Care staff (e.g., certified nursing assistants) develop intimate knowledge of each person they care for, how they respond to various types of care, and the impact of such responses on the care environment. We are examining multiple cases (six) to reflect the care landscape within Kentucky and are using maximum variation sampling to ensure diverse identities and care experiences are represented. Data sources from each case are: (a) two interviews, (b) six weekly journal entries, (c) one site observation, and (d) publicly available facility information. Analysis involves rich case description, case‐wise thematic analysis, and cross‐case thematic analysis following Creswell’s (2013) data analysis spiral: (1) reading and memoing, (2) describing and interpreting data into codes and themes, (3) interpreting the data, and (4) representing and visualizing the data.

**Results:**

To have transferability of results, readers must be able to gain an in‐depth understanding of each case. Therefore, planned enrollment (January to April 2024) is 6 care staff from non‐profit and for‐profit facilities across rural, suburban, and urban areas of Kentucky. Active data collection begins in February, and results are expected by July 2024.

**Conclusion:**

The care needs of persons with dementia are high and require nonpharmacological options to maximize well‐being. Identifying ways to reliably document clinically meaningful outcomes without burdening caregivers is crucial to advance evidence‐based care. Results will provide insights into these challenges and transfer to the broader dementia care landscape.

